# Effects of Kinesiology Taping on Shoulder Posture and Peak Torque in Junior Baseball Players with Rounded Shoulder Posture: A Pilot Study

**DOI:** 10.3390/life10080139

**Published:** 2020-08-06

**Authors:** Hyeong-geun Yun, Jung-Hoon Lee, Im-Rak Choi

**Affiliations:** 1 Department of Biomedical Health Science, Graduate School, Dong-Eui University, Busan 47340, Korea; heyhyoung@naver.com; 2 Department of Physical Therapy, College of Nursing, Healthcare Sciences and Human Ecology, Dong-Eui University, Busan 47340, Korea; 3 Integrated Physical Medicine Institute, Dong-Eui University, Busan 47340, Korea; 4 Department of Rehabilitation Therapy Team, Sports Exercise Therapy Center, Good Samsun Hospital, Busan 47007, Korea

**Keywords:** rounded shoulder posture, baseball player, kinesiology tape, peak torque

## Abstract

Rounded shoulder posture (RSP) causes an imbalance of the adjacent joints due to the malalignment of the shoulder joint, and thus affects the strength of the muscles surrounding the shoulder. This study aimed to investigate the effect of rounded shoulder taping (RST) on shoulder posture and muscle strength in junior baseball players. Nineteen junior baseball players participated in the study, which used a crossover design at an interval of 1 week. The participants were randomized to receive rounded shoulder taping (RST) and sham kinesiology taping (SKT) using kinesiology tape. RSP was measured using two 12-inch combination squares, and shoulder peak torques were measured by isokinetic equipment. The results showed that RST led to significant changes in RSP (*p* < 0.05), but no significant changes were observed with SKT (*p* < 0.05). RST led to significant changes in the peak torques of external rotation and internal rotation of the shoulder (*p* < 0.05), but no significant changes were observed with SKT (*p* < 0.05). These results suggest that RST could help to correct RSP and improve peak torque of external and internal rotation of the shoulders of junior baseball players with RSP.

## 1. Introduction

For baseball players, effective use of the muscles surrounding the scapula is a critical factor in maintaining the mobility and stability of the shoulder [1,2]. The motion of overhead throwing demands frequent use of the muscles surrounding the scapula, and powerful rotation of the scapula is required [3]. Accordingly, the changed position of the scapula after overhead throwing is related to shoulder injury [3]. Baseball players who often do overhead throwing repeat the step of cocking, wherein the scapula is retracted, as well as the step of acceleration, wherein the scapula is protracted [4]. Scapular protraction causes sustained stretch of the scapula retractor muscles, with a fall in scapula retractor muscle strength being reported to cause rounded shoulder posture (RSP) [5,6].

RSP has been reported to occur because of the stress imparted on the muscles due to repeated movements and sustained posture [7,8]. RSP increases the kyphosis of upper thoracic vertebrae and the lordosis of the cervical vertebrae [8], while causing the scapula to show anterior tilt, downward rotation, and protraction [9]. RSP also leads to increased muscle tension in the levator scapulae and upper trapezius, tightness and weakness in the pectoralis minor and pectoralis major, and dysfunction of the glenohumeral joint due to an imbalance caused by weakness due to stretching of the middle and lower trapezius [10].

Treatments for RSP include protraction of the pectoralis minor [11] and stabilization of the muscles surrounding the scapula [12]. In previous studies wherein adult patients were requested to perform pectoralis minor stretching, orthosis use, and scapula exercises, the degree of RSP decreased, while the scapular exercises was found to have led to high levels of activation in the lower trapezius and serratus anterior [13].

Kinesiology tape, which is one of the various interventions used for posture correction, is widely used in the treatment of musculoskeletal disorders for athletes and the general population, increases proprioceptive sensation [14], maintains postural stability [15], provides joint stability, and improves muscle strength [16,17,18]. When kinesiology tape was used in patients with scapular depression syndrome, scapular alignment and upper trapezius tenderness were shown to have improved [19]. Additionally, it also improved lower shoulder pain, as well as muscle strength for the upper and lower trapezius, as well as the serratus anterior muscles when it was used in patients with scapular downward rotation [20]. When the kinesiology tape was applied to athletes with scapular dyskinesis, upper trapezius activity decreased, and scapular kinematics, such as scapular posterior tilt and upward rotation, improved [21]. Application of the kinesiology tape to the upper trapezius was more effective in pain relief than postisometric muscle relaxation intervention [22]. Furthermore, when it was applied to the pelvis of patients with low back pain having sacroiliac joint dysfunction and an increased sacral horizontal angle, the pelvic anterior tilt and pain were reduced [23]. When it was used for individuals with pelvic posterior tilt, the pelvic tilt was found to have been immediately corrected [24].

In previous studies, applying kinesiology tape in seated workers with RSP produced immediate correction of RSP [25,26]. Nonetheless, there is a general lack of studies regarding the immediate effects of Rounded Shoulder Taping (RST) using kinesiology tape on junior baseball players with RSP in terms of RSP correction and peak torque. The present study thus aimed to investigate the immediate effects of applying RST and sham kinesiology taping (SKT) on RSP correction and shoulder peak torque in junior baseball players with RSP.

## 2. Materials and Methods

### 2.1. Study Design

**Figure 1:**
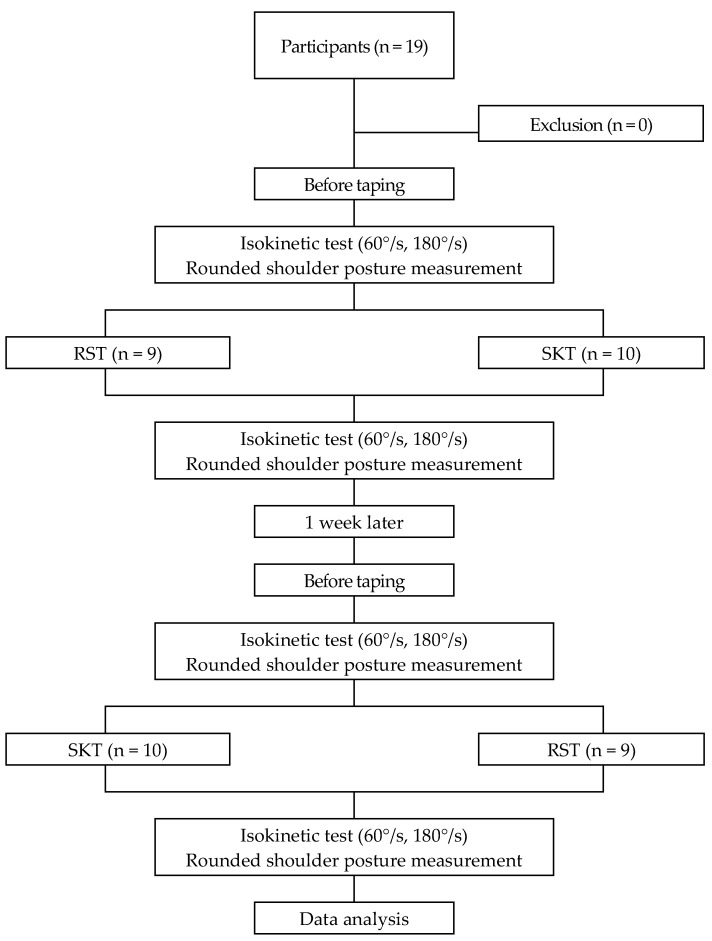
Flow diagram for the study. RST, Rounded Shoulder Taping; SKT, Sham Kinesiology Taping.

This study was a cross-over study carried out on 19 subjects who consented to participation, with IRB approval done by the Dong-Eui University Institutional Review Board (DIRB-201811-HR-E-39). To examine the effects of kinesiology taping on RSP correction and shoulder peak torque in junior baseball players with RSP, the participants were first given counterbalancing in a crossover manner so as to prevent execution effects, fatigue effects, and order effects of RSP and SKT. The application order of RST and SKT was randomized using computer-generated random numbers. Prior to applying RST or SKT, RSP was measured using the two 12-In combination square, while shoulder peak torque was measured using an isokinetic equipment. After a 10 min break, kinesiology tape was used to apply RST and SKT one at a time, and RSP and shoulder peak torque were measured once again immediately afterward. RSP and shoulder peak torque were measured by the same investigator blinded to the method of kinesiology taping. After a week, RSP and shoulder peak torque were measured again using the two 12-in combination square and isokinetic equipment, respectively. After a 10 min break, the participants were given RST and SKT in the reverse order they were given in the previous week, and immediately afterwards, the RSP and shoulder peak torque were measured once again. A flowchart of the experimental design and procedures are given in Figure 1.

### 2.2. Participants

The participants were 19 junior baseball players with RSP. The inclusion criteria were as follows: (1) an individual who has not shown an abnormality in the musculoskeletal or neurological system in the past three months; (2) an individual who does not show limited shoulder pain or joint range of motion (ROM); (3) an individual without a history of contact dermatitis or abnormal skin reaction to the kinesiology tape; (4) an individual showing a ≥1 inch distance between the posterior and anterior of the acromion [8,27]; and (5) an individual who does not have previous experience with kinesiology tape application to avoid recognition of sham treatment.

### 2.3. Sample Size

After computation using G-power 3.1 (University of Dusseldorf, Dusseldorf, Germany) with effect size 0.8, significance level (α level) 0.05, and testing power 0.8, the sample size required for examining the effects of RST and SKT on RSP and shoulder peak torque were found to be 15 individuals. Considering the possibility of drop-outs, 19 subjects were recruited [28].

### 2.4. Kinesiology Tape Application

Both RST and SKT were performed by a physical therapist having experience with kinesiology taping of five years or more.

#### 2.4.1. Rounded Shoulder Taping

**Figure 2:**
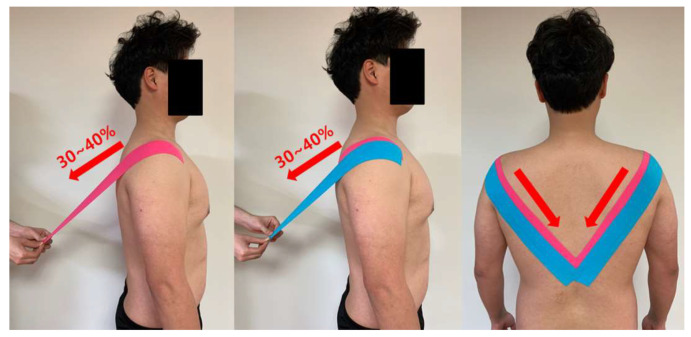
Rounded shoulder taping.

RST using kinesiology tape (BB TAPE, WETAPE Inc., Pyeongtaek, Korea) was performed while the subject was standing upright in a posture with the scapulae retracted, with the kinesiology tape applied from the anterior of the scapular acromion up to the spinous process of the 10th thoracic vertebra and stretched to 30–40% greater length [25,26,29]. To enhance the mechanical effect, the kinesiology tape was applied once again to the same area of previous application, but now with a 50% overlap and stretched to 30–40% greater length [25,26,29] (Figure 2).

#### 2.4.2. Sham Kinesiology Taping

**Figure 3:**
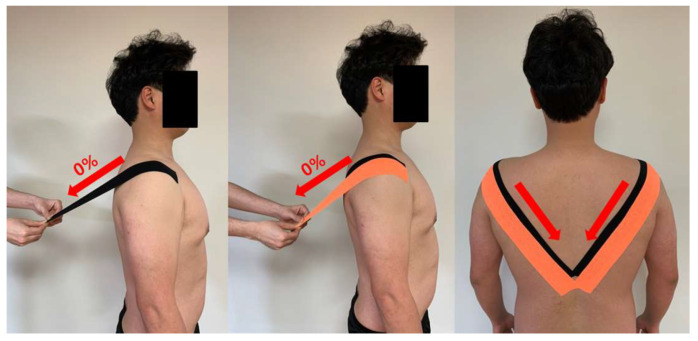
Sham kinesiology taping.

SKT was performed while the subject was standing upright in a posture with the scapulae retracted, with the kinesiology tape applied from the anterior of the scapular acromion up to the spinous process of the 10th thoracic vertebra without stretching. The kinesiology tape was applied once again to the same area of previous application, but now with 50% overlap and without stretching [25] (Figure 3).

### 2.5. Measurement

#### 2.5.1. Two 12-in Combination Square

**Figure 4:**
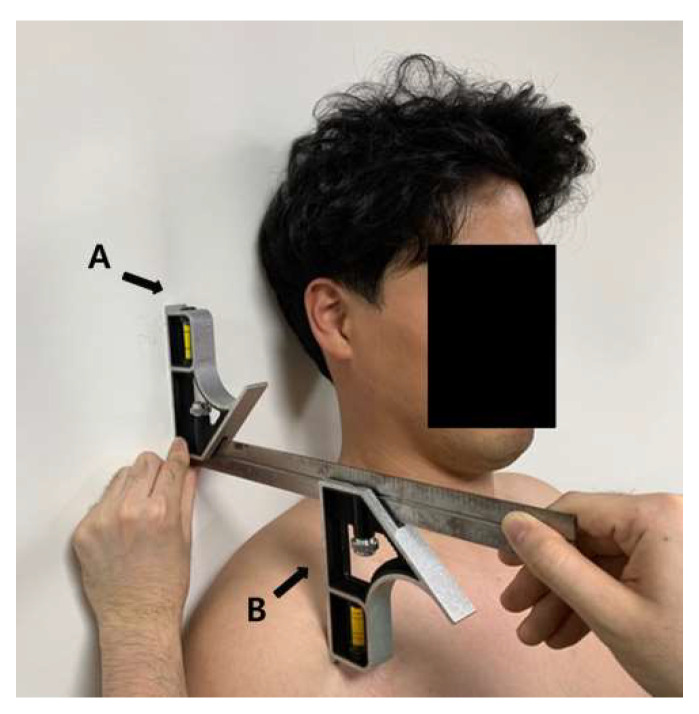
Rounded shoulder measurement equipment (two 12-in combination square). **A**: the first square in the two 12-in combination square; **B**: the second square in the two 12-in combination square.

To measure any immediate changes in RSP correction after kinesiology taping, the two 12-in combination square (Johnson Level & Tool manufacturing Co, Inc., Mequon, WI, USA) was used. The device is composed of two squares and a graduated ruler for measuring the change in distance between two points. While the subject was standing against a wall, the first square in the two 12-in combination square was placed parallel to the wall above the shoulder (Figure 4A). The second square was placed at the end of the anterior of the scapular acromion, and the distance between the wall and scapular acromion was measured so as to estimate the degree of RSP (Figure 4B) [27]. The distance between the wall and acromion process was measured three times and the mean value was used in subsequent analyses [30].

#### 2.5.2. Isokinetic Equipment

**Figure 5:**
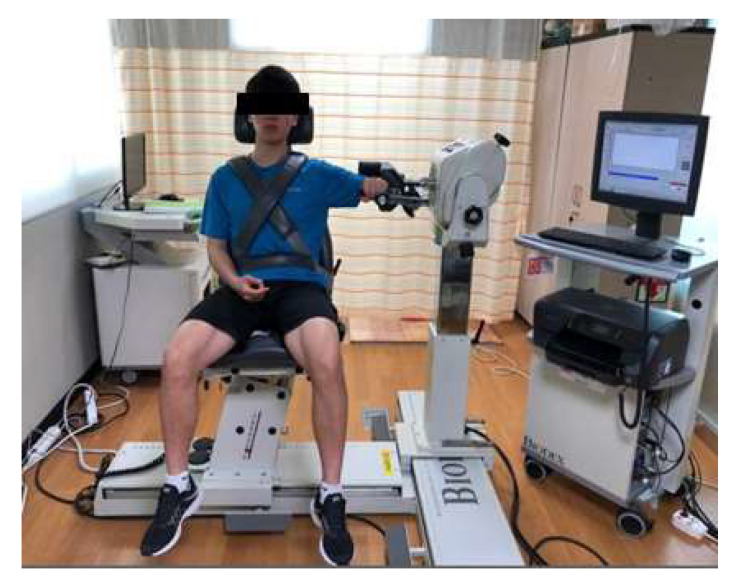
Isokinetic test equipment (Biodex).

To measure shoulder peak torque, isokinetic equipment Biodex (Biodex system 4, Biodex Medical System Inc., New York, NY, USA) was used (Figure 5). Biodex is a device that uses a concentric or eccentric mode to measure muscle strength or allow muscle training to be performed. The subject sat on the Biodex device with shoulders at 90° abduction, elbows at 90° flexion, and forearm pronation [31]. Muscle strength, which represents the maximal power against resistance, and power, which is related to the strength and movement speed [32] of external rotator and internal rotator muscles, were each measured five times. Muscle strength was measured at an angular velocity of 60°/s, and power was measured at an angular velocity of 180°/s [33]. For each measurement, the subject was given a 60 s break before peak torque values were taken [34]. Biodex defines the peak torque as the highest point along the measured curve during the movement, and the device reliability and validity were 0.82–0.95 [35,36].

### 2.6. Statistical Analysis

For data treatment and analyses, SPSS 18.0 (IBM Corp., Armonk, NY, USA) was used. When the normality test was carried out using the Shapiro-Wilk and Kolmogorov-Smirnov tests, the measured date by using Biodex were distributed normally at *p* > 0.05. Descriptive statistics were used for the general characteristics of the subjects, while a paired *t*-test was used to compare changes in RSP correction and shoulder muscle strength after RST and SKT. To compare changes in RSP correction and shoulder muscle strength between the two interventions, an independent *t*-test was carried out. The statistical significance level was set to 0.05.

## 3. Results

### 3.1. General Characteristics

All 19 subjects were male junior baseball players, with the dominant arm being the right arm and the left arm for 16 and 3 of them, respectively. The average age of the subjects was 17.33 ± 1.49 years, while the average height and weight were 174 ± 4.28 cm and 80.8 ± 12.92 kg, respectively (Table 1).

### 3.2. Changes in Shoulder Posture

When applying the RST and SKT each to junior baseball players with RSP, the distance in RSP was found to have immediately significantly decreased after RST (*p* < 0.05) (Table 2), while no significant change was observed in RSP distance after SKT (*p* < 0.05) (Table 2). The change in RSP before and after applying RST and SKT in junior baseball players with RSP showed no significant differences between the two interventions (*p* < 0.05) (Table 3).

### 3.3. Changes in Shoulder Peak Torque

After applying RST and SKT to junior baseball players with RSP, the peak torque at angular velocity 60°/s and 180°/s were found to have both immediately significantly increased after RST (*p* < 0.05) (Table 4), while no significant change was observed in the peak torque after SKT (*p* > 0.05) (Table 5). The change in peak torque before and after applying the RST and SKT in junior baseball players with RSP showed significant differences between the two interventions (*p* < 0.05) (Table 6).

## 4. Discussion

### 4.1. Relationship between Kinesiology Taping and RSP

The results in this study showed that when RST and SKT using kinesiology tape were applied to junior baseball players with RSP, only RST led to a significant reduction in RSP. In a previous study involving scapula correction in female handball players, kinesiology tape was applied by being stretched from the coracoid process past the upper trapezius to the lower trapezius, leading to improvement in the posterior tilt and upward rotation of the scapula through the mechanical effect of the kinesiology tape [37]. When kinesiology tape was stretched and applied to overhead athletes with scapular dyskinesis, mechanical correction of the abnormal scapular movement was observed [38]. Applying kinesiology tape to individuals with RSP showed that when the tape was stretched and applied to both shoulders while both scapulae were retracted, the shoulders of the individuals tried to return to the previous RSP but the kinesiology tape, due to its reduced elasticity, conferred resistance to RSP—thus creating an immediate correction effect [25]. In sedentary workers with RSP, the kinesiology tape was stretched and applied to both shoulders continuously for one month, and as a result, reduced RSP and recovery from the dominant upper back pain were observed [26]. In this study, likewise, 30–40% stretching and application of kinesiology tape while both scapulae were retracted led the shoulders to initially try to return to the previous RSP, but the increased tension of stretched kinesiology tape resisted RSP. The elasticity of the kinesiology tape also promoted a retracted posture of the shoulders, which is thought to have reduced RSP [26].

### 4.2. Relationship between Kinesiology Taping and Shoulder Peak Torque

When RST and SKT were applied to junior baseball players with RSP, significant increases were observed after RST at angular velocities of 60°/s and 180°/s and in the shoulder torque for both external and internal rotation. Muscle strength is under the influence of actin and myosin alignment based on muscle fiber length [31]. Maximal strength is produced when the length of the muscle fiber is optimal in the context of length-tension relation [39]. A further increase or decrease in the length between the muscle fiber actin and myosin would lead to a reduction in the number of cross bridges, thus causing the produced active strength to fall in its magnitude despite maximum effort [31]. Muscle activation is reduced in RSP as the scapula protracts, rotates downward, and anteriorly tilts, which altogether lead to weakness due to stretching of the middle and lower trapezius, as well as due to tightness of the pectoralis major and pectoralis minor, in addition to a reduction in the length of the serratus anterior [10,40,41].

Applying kinesiology tape while both scapulae were retracted for RSP correction led to immediate retraction of the protracted scapula and elongation of the shortened pectoralis muscle [25]. As a result, the humerus head was moved to the center of the glenoid fossa, leading to effects on shoulder rotator muscles and length tension while providing stability to the scapula, which together led to an increase in shoulder rotator cuff strength [4,42]. When kinesiology tape was applied for scapula correction in asymptomatic volleyball players, a decrease in acromiohumeral distance, as well as increased ROM of shoulder external and internal rotations were observed, with the scapula correction leading to a significant increase in the strengths of shoulder external and internal rotations [42]. The scapula of an individual performing an overhead motion plays a crucial role in energy transfer when moving from a proximal to distal direction [4], with the position of the scapula having an effect on the strength produced upon joint rotation of the shoulder [43]. In this study, likewise, applying the kinesiology tape in junior baseball players with RSP led to the correction of the scapula to the neutral position, which influenced the length and tension of the shoulder rotator cuff such that the muscles could produce maximal strength. In addition, as scapula stability increased and posture improved, energy transfer became more efficient, thus leading to increased muscle strength at 60°/s and increased peak torque at 180°/s.

### 4.3. Limitations

There are limitations to this study. First, since the study involved junior baseball players, the findings cannot be generalized to all sports players. Second, only the immediate effects of the kinesiology taping were compared, and thus, long-term effects have not been verified. Third, measurement of shoulder peak torque focused only on external and internal rotations, with changes in the peak torque based on various shoulder movements not estimated in this study. Fourth, this study was not registered on the clinical trial platform. Finally, the standard deviation was greater than the mean due to the small number of participants. Therefore, further studies investigating the long-term effects of RST using kinesiology tape on RSP reduction and muscle strength improvement for various shoulder movements in athletes of varying age groups and a larger number of subjects are warranted.

## 5. Conclusions

In this study, RST using kinesiology tape after 30–40% stretching was applied to junior baseball players with RSP, which led to immediate reductions of RSP and increased peak torques for both shoulder external and internal rotations. The use of the RST is suggested for immediate RSP reduction and improvement of the peak torques of shoulder external and internal rotations in junior baseball players with RSP. However, further studies should be conducted on many baseball players with round shoulders in order to present clinical effects on round shoulder correction and muscle strength.

## Figures and Tables

**Table 1:** General characteristics (n = 19).

Variables	Participant (n = 19)
Gender (male/female)	19 (100%)/0 (0%)
Dominant (right/left)	16 (84%)/3 (16%)
Age (years)	17.42 ± 1.46 ^†^
Height (cm)	175.10 ± 4.45
Weight (kg)	80.05 ± 11.98

^†^ mean ± SD.

**Table 2:** Changes in shoulder posture after kinesiology taping.

Variables	Pre-Taping	Post-Taping	*p*
RST (cm)	14.88 ± 1.63 ^†^ (15.25, 1.75)	14.19 ± 1.63 (13.00, 2.25)	0.00 *
SKT (cm)	14.88 ± 1.63 (15.25, 1.75)	14.81 ± 1.62 (15.25, 2.00)	0.09

* *p* < 0.05, ^†^ mean ± SD (median, quartiles range), RST, rounded shoulder taping; SKT, sham kinesiology taping.

**Table 3:** Comparison of the amount of changes in shoulder posture between the two kinesiology taping methods.

Variable	RST	SKT	*p*
Distance (cm)	0.68 ± 0.18 ^†^ (0.75, 0.25)	0.06 ± 0.16 (0.00, 0.25)	0.00 *

* *p* < 0.05, ^†^ mean ± SD (median, quartiles range), RST, rounded shoulder taping; SKT, sham kinesiology taping.

**Table 4:** Changes in shoulder peak torque after rounded shoulder taping (RST).

Variables		Pre-Taping	Post-Taping	*p*
External rotation (Nm)	60°/s	27.70 ± 9.09 ^†^ (26.90, 16.80)	29.26 ± 8.63 (28.90, 15.00)	0.00 *
	180°/s	23.93 ± 8.17 (23.20, 14.90)	25.25 ± 8.30 (24.60, 12.70)	0.02 *
Internal rotation (Nm)	60°/s	39.57 ± 11.95 (39.70, 15.00)	42.10 ± 10.80 (41.30, 13.30)	0.02 *
	180°/s	36.80 ± 10.29 (38.20, 19.70)	39.05 ± 10.65 (41.70, 18.80)	0.01 *

* *p* < 0.05, ^†^ mean ± SD (median, quartiles range).

**Table 5:** Changes in shoulder peak torque after sham kinesiology taping (SKT).

Variables		Pre-Taping	Post-Taping	*p*
External rotation (Nm)	60°/s	27.95 ± 8.81 ^†^ (24.90, 15.00)	28.13 ± 9.21 (25.90, 13.40)	0.72
	180°/s	24.36 ± 7.92 (23.80, 14.50)	24.21 ± 8.04 (22.40, 12.00)	0.38
Internal rotation (Nm)	60°/s	38.88 ± 11.77 (39.10, 17.60)	37.77 ± 9.22 (39.30, 13.20)	0.60
	180°/s	35.92 ± 9.91 (37.40, 15.50)	35.46 ± 9.65 (36.90, 18.60)	0.55

* *p* < 0.05, ^†^ mean ± SD (median, quartiles range).

**Table 6:** Comparison of the amount of changes in shoulder peak torque between the two kinesiology taping methods.

Variables		RST	SKT	*p*
External rotation (Nm)	60°/s	1.56 ± 1.66 ^†^ (1.50, 2.40)	0.18 ± 2.21 (0.30, 2.80)	0.03 *
	180°/s	1.31 ± 2.36 (1.70, 2.10)	−0.15 ± 1.26 (0.50, 2.30)	0.02 *
Internal rotation (Nm)	60°/s	2.52 ± 4.56 (2.00, 6.60)	−1.10 ± 5.42 (0.30, 7.90)	0.03 *
	180°/s	2.25 ± 3.54 (2.40, 2.60)	−0.45 ± 3.29 (1.70, 4.30)	0.02 *

* *p* < 0.05, ^†^ mean ± SD (median, quartiles range), RST, rounded shoulder taping; SKT, sham kinesiology taping.
